# Infra-3DRC-FusionNet: Deep Fusion of Roadside Mounted RGB Mono Camera and Three-Dimensional Automotive Radar for Traffic User Detection

**DOI:** 10.3390/s25113422

**Published:** 2025-05-29

**Authors:** Shiva Agrawal, Savankumar Bhanderi, Gordon Elger

**Affiliations:** 1 Institute of Innovative Mobility (IIMo), Technische Hochschule Ingolstadt, 85049 Ingolstadt, Germany; savankumar.bhanderi@thi.de (S.B.); gordon.elger@thi.de (G.E.); 2 Fraunhofer IVI, Applied Center Connected Mobility and Infrastructure, 01069 Dresden, Germany

**Keywords:** artificial intelligence, camera, deep learning, data processing, object detection, perception, radar, roadside-mounted sensors, sensor data fusion, smart infrastructure

## Abstract

Mono RGB cameras and automotive radar sensors provide a complementary information set that makes them excellent candidates for sensor data fusion to obtain robust traffic user detection. This has been widely used in the vehicle domain and recently introduced in roadside-mounted smart infrastructure-based road user detection. However, the performance of the most commonly used late fusion methods often degrades when the camera fails to detect road users in adverse environmental conditions. The solution is to fuse the data using deep neural networks at the early stage of the fusion pipeline to use the complete data provided by both sensors. Research has been carried out in this area, but is limited to vehicle-based sensor setups. Hence, this work proposes a novel deep neural network to jointly fuse RGB mono-camera images and 3D automotive radar point cloud data to obtain enhanced traffic user detection for the roadside-mounted smart infrastructure setup. Projected radar points are first used to generate anchors in image regions with a high likelihood of road users, including areas not visible to the camera. These anchors guide the prediction of 2D bounding boxes, object categories, and confidence scores. Valid detections are then used to segment radar points by instance, and the results are post-processed to produce final road user detections in the ground plane. The trained model is evaluated for different light and weather conditions using ground truth data from a lidar sensor. It provides a precision of 92%, recall of 78%, and F1-score of 85%. The proposed deep fusion methodology has 33%, 6%, and 21% absolute improvement in precision, recall, and F1-score, respectively, compared to object-level spatial fusion output.

## 1. Introduction

Roadside-mounted smart infrastructure-based environment perception, especially traffic or road user detection, plays a vital role in enhancing the safety of people on the road, optimizing the traffic flow in the cities, and assisting nearby passing vehicles by providing extra information and relevant warnings about critical conditions on the road. The field of view of vehicle sensors is often limited due to occlusion, lower mounting, and low distant detection capabilities. But infrastructure units are equipped with sensors at high elevations and suffer low occlusions [1,2,3] as compared to vehicle sensors. The robust perception information of infrastructure units is then sent to nearby vehicles in real time for better decision making [1,4,5].

In recent years, RGB (red, green, and blue) camera-based infrastructure units have been applied to surveillance, traffic monitoring, vehicle counting, etc. [6]. However, these cameras are highly prone to bad light and weather conditions, which drastically decrease their detection capabilities [7]. Moreover, the detections from the camera are in 2D image space, which does not map the real 3D physical world. For many applications, the detection and recognition of traffic users in a 3D vector space is necessary. The solution to all these shortcomings is to use radar or lidar sensors together with a camera. Compared to a lidar sensor, a radar sensor is very robust in different light and weather conditions, has low cost, and provides accurate and long-range depth and velocity information, which makes it a very good complementary sensor to use with a camera. By fusing radar and camera data, one can harness both sensors’ benefits and overcome their limitations jointly [8].

However, in the most widely used decision-level fusion (includes object-level fusion and track-level fusion) [9], the camera and radar are individually and independently processed to generate objects, and then the processed information is fused at a later stage (also referred to as late fusion). But in bad weather and light conditions, cameras cannot detect very well independently, and hence their contribution to such fusion techniques drastically reduces the final object detection capabilities. The solution to this problem is to fuse the data intelligently using deep neural networks from both sensors at an early stage [8,9]. By fusing at an early stage, it is possible to use complete data provided by both sensors and hence, in challenging environmental conditions, even when one sensor measurement degrades, measurements from another sensor help to improve the final output. In early-stage fusion, data from two sensors can be fused either at a very early stage, generally referred to as data-level fusion, or can be fused after extracting some intermediate features, commonly referred to as feature-level fusion [8]. Data-level fusion merges data from two sensors without any processing; hence, it requires very accurate spatial and temporal alignment between the data from both sensors. It is computationally expensive and may add noise to the outcome, even if one sensor input has high noise. Feature-level fusion has a good trade-off between data-level and decision-level fusion in terms of computation, speed, and data usage [9].

There has been some research carried out to fuse camera and radar data using deep neural networks but their work is limited to only vehicle-based perception and the available research in smart infrastructure-based road user detection [10,11,12,13,14,15] is limited to some or other forms of decision-level fusion only (best known to the author). Even though the aim of both vehicle-based perception and smart infrastructure-based perception is to detect and classify various road users, there are many differences in the overall perception pipeline. First and foremost, infrastructure sensors are mounted on roadside poles, bridges, buildings, etc., where the mounting height varies approximately from 4 to 12 m from the ground whereas sensors (especially cameras and radars) in vehicles are mounted at a height of approximately from 0.3 to 1.5 m only. This drastic difference in mounting height leads to a completely different field of view and hence different data distribution of radar and camera during data collection. In smart infrastructure, sensors have a wide view of the road and road users due to high elevation, which reduces occlusions to a great extent as compared to low-level mounted vehicle sensors. Also, due to differences in data distribution and view angle, the datasets generated using vehicle sensors are not usable for research in infrastructure-based perception. Further, sensors in infrastructure setups are always static, and hence, for a given deployed system, the field of view of the static environment (road lanes, structures, curves, position of traffic lights, street lights, etc.) remains the same. Hence, this knowledge of location is used to pre-process radar and camera data to remove unwanted background or noise points, and also helps to further optimize the final 3D object detection by using geographical knowledge. However, in vehicle-based perception, sensors are in motion, and hence, such techniques are not helpful. During bad light and weather conditions, vehicle-based cameras have better visibility due to illumination from vehicle headlights, but in smart infrastructure, illumination from street lights is highly uneven and creates many dark zones within the sensor field of view that are required to be considered for consistent road user detection. There are also cases where street lights are not installed in some areas, which adds further challenges to the infrastructure-based detection algorithms. Vehicle-based sensor fusion algorithms have limitations due to embedded memory and space, but in a smart infrastructure setup, it is possible to use high computing devices because they are easily installable on roads and open doors to research and development opportunities with computationally expensive algorithms to obtain high performance. Considering all these reasons, it is evident that the sensor data distribution from high elevation, static sensor data pre-processing, dataset requirements, challenges in low light and bad weather conditions, and knowledge of fixed environment jointly influence the development of deep fusion algorithms for object detection for smart infrastructure-based perception. Furthermore, due to these differences, the deep neural network models developed for vehicle-based platforms are not directly usable for smart infrastructure-based perception. However, the research in the vehicle domain proves that low-level fusion of radars and cameras enhances object detection, and hence, it is wise to conduct research using roadside-mounted smart infrastructure-based sensors.

With this motivation, in this work, a novel deep fusion architecture is proposed that fuses RGB mono-camera images and 3D radar point cloud data using a deep learning network to generate enhanced object detection of various road users in the 3D vector space (also referred to as ground plane) of the smart roadside infrastructure unit. This deep neural network is named *Infra-3DRC-FusionNet.* Five different road user categories, namely person, bicycle, motorcycle, car, and bus, are considered, and evaluations in different light and weather conditions are performed. The proposed fusion methodology is developed in a way that, as per the requirement of the application, one can independently generate point objects as well as 3D extended objects in the ground plane of the smart roadside infrastructure unit using the predicted output of the Infra-3DRC-FusionNet. It si worth noting that this work focuses on enhancing object detection, which is only frame-wise output. Generally, object detection is the first stage in any perception pipeline that is followed by tracking (multi-frame dependent output). But tracking is not part of this work.

This paper is structured as follows: Section 2 provides insights into available state-of-the-art work, and Section 3 briefly describes the measurement setup, the process of data collection, dataset generation, and provides an overview of the training and validation sets. It also briefly describes the ground truth data. Section 4 describes the deep neural network architecture proposed in this work for camera and radar fusion and then describes each part of the network in detail. Section 5 provides details of the training of the network, experiments performed, and their results, along with some discussion. At last, a conclusion is provided.

### Contributions

The main contributions are as follows:

- A novel deep fusion architecture is proposed to fuse RGB mono-camera image and 3D radar point cloud data to enhance frame-wise object detection in the 3D ground plane of the smart infrastructure-based sensor setup.
- A 3D radar-based region proposal generator is developed to enhance object detection in bad light and weather conditions by taking inspiration from [16].
- Various experiments are conducted both at the feature level (using separate backbones for each sensor) and low level (combining all six channels of camera and radar in one backbone) to find the best performance of the model.
- The output of the model is validated with Lidar ground truth data, and it is benchmarked with four different variants of frame-wise object-level (high-level) spatial fusion for various environmental conditions.

## 2. Related Work

Work of [17,18,19,20,21,22,23,24,25,26,27,28,29,30,31,32,33,34,35,36] was carried out to fuse data using deep neural networks for the vehicle-mounted radar and camera sensors. However, the author’s work in [17,18,19,20,21,22,23,24,25,26,27,28,29] is limited to object detection in the 2D image plane of the camera in the form of 2D bounding boxes. The process of the 2D image plane to the 3D ground plane is an irreversible problem in computer vision without the help of depth information from other sensors; hence, these approaches are not sufficient to detect and classify road users in 3D vector space (also known as the 3D ground plane of the infrastructure unit). The authors of [30,31] have fused radar and camera data using low-level fusion, but their work is limited to the detection of only cars. Other road users, especially VRUs (vulnerable road users), are not considered in their work.

Authors in [32] use centernet to first perform 3D object detection using only a camera and then use radar to further enhance the quality of output. However, during bad light and weather conditions, when the camera cannot see properly, this approach is not usable, and their work is also limited to 2D radars. The authors of [33] fused radar and camera data in BEV (bird-eye-view) by encoding the semantic information of the camera to the BEV plane using semantic-point-grid. However, their approach uses a pre-trained semantic segmentation model to extract semantic features of each road user from camera images, but it is unclear from the paper how these features are extracted when the object is hardly visible in camera images. The authors of [34] use voxelization of radar points for fusion with the camera, but their work is limited to 2D radar sensors. Moreover, they have used multi-frame accumulation of radar data to increase the point cloud, and also radar data are replicated at each point five times at its respective height position of 0.2 m and 0.5 m above and below. This is very specific to vehicle-based sensor data distribution and not usable for high-elevation infrastructure sensors. The authors of [35] use their developed backbone architecture to fuse radar and camera for 3D object detection in the camera image plane. However, the work is highly convoluted, and it is unclear from the paper whether the final 3D bounding boxes are transferable to the ground plane or only restricted to the 2D image plane. The work of [35] is very recently developed and uses a two-stage approach to enhance object detection using cameras and radars for vehicles.

The available research in smart infrastructure-based road user detection is limited (best known to authors) to some or other forms of decision-level fusion [10,11,12,13,14,15]. The authors of [10] fused radar tracks and camera tracks together by joint calibration. The authors of [11] focused more on improving the trajectory prediction of road users by combining the tracking in image coordinates with a Kalman filter performing in road coordinates. The authors of [12] implemented a state-of-the-art object-level fusion algorithm together with tracking, where YOLOv3 (you only look once version 3) is used as the camera detector and DBSCAN (density-based spatial clustering of applications with noise) for the radar-based object detector. Then, associate objects from each sensor in space and time to generate associated objects. The work described in [13] used regions of interest from radar and camera independently, matched them for high confidence, and then classified different types of cars using a small CNN (convolutional neural network). The authors of [14,15] fused camera and radar at the object level for the infrastructure-based perception of various traffic users.

## 3. Measurement Setup and Dataset Generation

**Figure 1:**
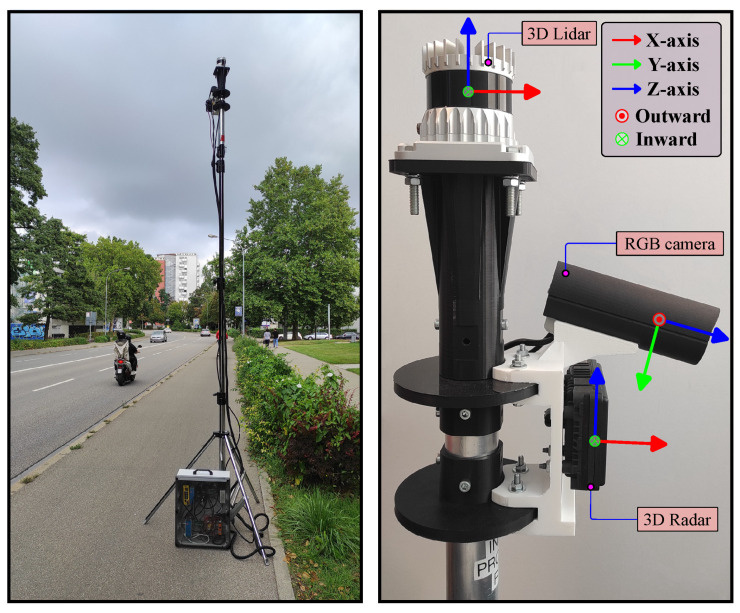
Smart infrastructure-based multi-sensor setup [37] used in this work. (**Left**) The setup is placed on one side of the road for data collection at a height of 3.5 m. (**Right**) Close view of the sensors along with their coordinate frames.

The smart infrastructure-based multi-sensor setup used in this work is shown in Figure 1. It consists of an RGB mono camera, 3D automotive radar, and a 3D automotive lidar sensor. Among them, RGB mono camera and 3D automotive radar sensors are used for deep fusion in this work, while the lidar sensor in the setup is used for joint calibration, mapping, and ground truth generation to validate the output of the proposed fusion methodology.

Hardware development of the smart infrastructure sensor setup is shown in Figure 1. The process of multi-sensor extrinsic calibration, data collection, and data labeling to generate a dataset for this work was previously published by our research group. Sections 4 and 5 of [4] provide details of the hardware, including sensors, electrical design, and mechanical design of this infrastructure setup. Ref. [38] provides in-depth information on the multi-sensor extrinsic calibration methodology for this setup. Similarly, the data collection process using software-based time synchronization and robot operating system (ROS) is described in Section 3 of [37].

To train the proposed Infra-3DRC-FusionNet, a large amount of labeled data is required. To avoid a high amount of manual work for labeling the complete dataset that comprises 7575 frames from an RGB mono camera and a 3D automotive radar sensor, a semi-automatic labeling methodology has been developed and published previously by [37]. In this approach, the cases where objects are easily detected by the camera are annotated automatically, while all the other difficult cases where the camera cannot detect due to bad light or weather conditions are annotated manually. For each pair of camera and radar images, the outcome from this methodology provides a list of 2D bounding boxes in the image plane, object category, and one or more radar points associated with each object. It is worth noting that in [37], six road user categories were used for labeling the data; however, the road user category “GROUP” is not included in this work because initial experiments with this category resulted in ambiguous outputs and conflicts between person, bicycle, and group categories.

### 3.1. Training and Validation Dataset

The complete dataset comprised 7575 frames of camera and radar data (time synchronized and calibrated) that are further split into approximately 70% and 30% as train and validation sets, respectively.

The complete dataset is collected at two different locations in the city of Ingolstadt, Germany. One location is a straight multi-lane bidirectional road (as shown on the left side of Figure 1), and another location is a pedestrian crossing junction with traffic lights and curved roads. Data are collected from various light conditions, such as early morning, daylight, twilight, and night, and various weather conditions, such as clear sky, cloudy, moderate rain, and heavy rain. Due to some constraints during data collection, data from snowfall and dense fog is not included. Table 1 provides instance-wise and frame-wise statistics of the training and validation sets. Furthermore, each set is divided into easy and difficult frames where the term “easy” refers to the data where the camera detects road users while “difficult” refers to the frames collected in adverse environmental conditions where due to bad light and/or weather conditions, the camera is not able to detect road users. From Table 1, it is evident that approximately 40% of frames in the total dataset are from adverse environmental conditions and 60% are from good environmental conditions.

In the city of Ingolstadt, the usage of motorcycles is quite low; therefore, very low instances were recorded in the dataset. Hence, augmentation is used to increase the total instances by almost five times. In this augmentation, only the brightness of the camera image is changed to different levels, and the corresponding radar pseudo image is not modified. As the proposed fusion methodology jointly uses camera and radar data, augmentation techniques such as cropping, flipping, zooming, etc., are not feasible because all these techniques change the positions of one or more road users from the original image results in data misalignment between corresponding camera image and radar point cloud data. The total instances of motorcycles given in Table 1 are calculated after augmentation. There is still an unbalance in the instances of various road users, which is taken care of during the training by assigning appropriate weights to each object category.

### 3.2. Ground Truth Data

Ground truth data comprised 28 scenes that are recorded separately in different light and weather conditions using the infrastructure setup of Figure 1 for all five road users. Table 2 highlights the statistics of these data. Each scene comprised sequentially recorded frames (80 to 150 frames) of synchronized (at 10 Hz) and calibrated lidar, radar, and camera data. Among them, lidar frames are manually labeled with 3D-oriented bounding boxes within the camera field of view by an external company and act as ground truth data for evaluating the performance of the proposed deep fusion model. The corresponding camera and radar frames of each scene are used as input to the deep fusion model to predict the road users. This ground truth data are also referred to as test data in the later part of this article.

In Table 2, apart from seven scenes recorded on the day with clear sky, all other scenes have poor light and/or bad weather conditions that help to validate the performance of the deep fusion network properly. Furthermore, the scenes recorded in “night” include different illuminations of light, ranging from twilight to night. Similarly, scenes recorded in “rain” include light, moderate, and heavy rain.

## 4. Methodology

**Figure 2:**
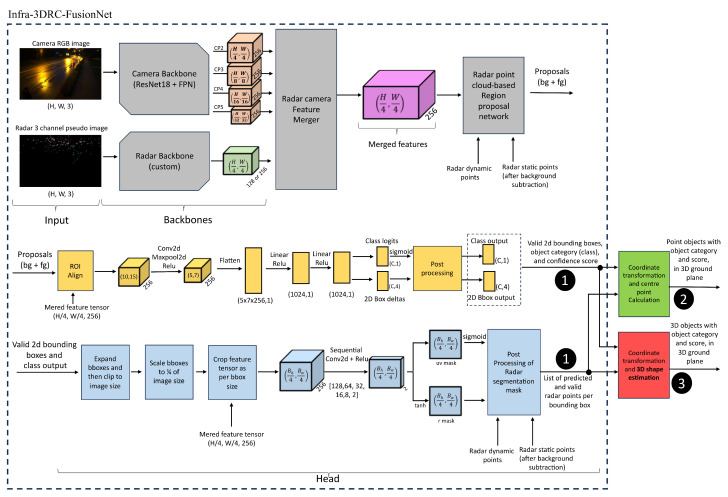
High-level architecture of the proposed Infra-3DRC-FusionNet.

The proposed object detection methodology in this work fuses calibrated and time-synchronized RGB camera image data and 3D radar point cloud data using a proposed novel deep learning architecture (named as Infra-3DRC-FusionNet) as shown in Figure 2. The prediction output from the model comprised 4 different information about each detected road user. The object category (person, bicycle, motorcycle, car, or bus), confidence score, 2D bounding box in the image plane, and the list of associated radar points. This output interface is marked as output 1 in Figure 2. This output is fed into a 3D point object generation block to obtain 3D point objects along with the corresponding object category and the confidence score of all the detected road users in the ground plane of the smart infrastructure setup (marked as output 2 in Figure 2). Independently, the predicted output is fed into a 3D shape estimator that generates 3D extended bounding boxes along with object category and confidence score in the ground plane of the smart infrastructure setup for various road users. This is marked as output 3 in Figure 2. In this paper, the model’s performance is evaluated at outputs 1 and 2. Details of 3D shape estimation and evaluations at output 3 will be presented and published separately in the future.

At first, the RGB camera image is fed into a camera backbone that comprises pre-trained state-of-the-art ResNet18 (Residual Network 18) [39] with FPN (Feature Pyramid Network) [40]. This neural network extracts features from the camera image at 4 different scales. Similarly, the 3D radar point cloud of the corresponding synchronized radar frame is converted into a three-channel radar pseudo image and then fed into a custom radar backbone to generate a feature tensor.

Features obtained from the radar and camera backbone are jointly fed into the radar-camera feature merger module to generate a merged feature tensor either by concatenation or element-wise addition of features. The merged features are fed into a radar point cloud-based region proposal generator that generates proposals for background (bg) and foreground (fg) classes by creating and processing anchors using 3D radar points. Then, these proposals, along with the merged feature tensor, are fed into the bounding box and class prediction head (blocks highlighted in yellow color in Figure 2). This head predicts object category (also known as class) with corresponding score (also known as confidence value) and 2D bounding boxes on the image plane.

Thereafter, only valid bounding boxes are filtered and given as input to the radar segmentation mask head (blocks highlighted in blue color in Figure 2). This head predicts spatial positions of the associated radar points for each bounding box in the image plane (as uv mask) and their distance estimation (as r mask). Both predicted outputs are fed into a post-processing block to generate the final associated radar points for each detected bounding box. All the predicted outputs of the model are then either fed to a 3D point object generator or a 3D shape estimator that generates 3D bounding boxes of each detected road user in a ground plane of a smart infrastructure-based sensor setup. Each major sub-part of the proposed deep learning architecture is explained in detail in this section.

### 4.1. RGB Camera Image

The RGB camera image given as input to the model is of size (1216, 1920) pixels, where each pixel value is in the range (0, 255) and it has 3 channels. To make it compatible with deep learning model computation, integer values of each pixel are converted into floating point values in range (0, 1) and then each channel is normalized using mean values [0.485, 0.456, 0.406] and standard deviation values [0.229, 0.224, 0.225] for red, green, and blue channels, respectively. These values are taken from the ImageNet [41] database and are very common to use in RGB image processing for deep learning models.

### 4.2. Three-Dimensional Radar Three-Channel Pseudo Image

The 3D radar point cloud of one frame contains multiple points where each 3D radar point has value range of (r) in meters, azimuth angle or horizontal angle (ϕ) in radians, elevation angle or vertical angle (θ) in radians, range rate or Doppler velocity $\left(v_{r}\right)$ in meters/second, and radar cross-section RCS (σ) in decibel/square meter. This is represented in the 3D physical space of the radar coordinate system.

This 3D space radar point cloud of one frame is converted into a three-channel RGB image-like format called a 3D radar pseudo image. This concept is inspired and adapted from the work of [18,19]. In this pseudo image, the 3D position of each radar point is encoded into corresponding pixel values (u,v) using a projection matrix and longitudinal distance *x*, longitudinal velocity $v_{x}$, and lateral velocity $v_{y}$ are encoded as *R*, *G*, and *B* channels, respectively, using appropriate encoding functions. The resulting pseudo image in this work is of size (1216, 1920, 3).

Statistical analysis was performed on the full dataset to decide which parameters to select for generating the radar pseudo image. From the distributions of $v_{x}$, $v_{y}$, and RCS, it is observed that unique patterns are exhibited in the distributions of $v_{x}$ and $v_{y}$ for each road user. In contrast, the distribution of RCS across all five road users is found to have very high variance and appears largely similar. To further substantiate this finding, the literature in the domain of RCS is referred to, and it is determined that RCS values are highly sensitive and fluctuate significantly due to factors such as the sensor’s view angle, the object’s surface area and type, and the distance between the object and the sensor. Consequently, the inclusion of RCS as a prominent channel in the radar pseudo-image may have introduced ambiguities in the output of the deep learning model. Therefore, RCS values are excluded from consideration. Their inclusion may be explored in future work if necessary.

Further, the process of radar pseudo-image generation consists of three steps.

#### 4.2.1. Spatial Position Encoding

Each radar point measurement is first converted from 3D polar coordinates to 3D Cartesian coordinates using Equation (1).

(1) $$\begin{array}{cc} x & =r\cos\left(\phi \right)\cos\left(\theta \right)\\ y & =r\sin\left(\phi \right)\cos\left(\theta \right)\\ z & =r\sin\left(\theta \right) \end{array}$$

A three-channel black canvas image with the same size as the camera image is created with all pixel values assigned to zero. This image acts as the base image for radar pseudo-image generation. Then, the (x,y,z) values of each 3D radar point from one frame are converted into corresponding pixel values (u,v), as described in Section 3 of [38]. In the base canvas, all the pixel values (u,v) that belong to projected radar points are assigned as 1 in all three channels, and all other spatial positions where no projected radar point is available are left as 0 only. This stage results in a three-channel binary image.

#### 4.2.2. Measurement Encoding

Now, the *R* channel of the pseudo-binary image is encoded with the radar point Cartesian distance *x*. The encoding function as given in Equation (2) that encodes the distance *x* from [0, 120] m to [−1, 1] linearly. These encoded values are then assigned as pixel values to the *R* channel of the pseudo image, where the value of 1 was assigned in the previous step.

(2) $$\begin{array}{cc} encoded\_x & =\frac{x}{60}-1 \end{array}$$

Longitudinal velocity $v_{x}$ and lateral velocity $v_{y}$ of each radar point is calculated from radar Doppler velocity using (3) and then values are encoded into *G* and *B* channels, respectively, using encoding function (4).

(3) $$\begin{array}{cc} v_{x} & =v_{r}\cos\left(\phi \right)\cos\left(\theta \right)\\ v_{y} & =v_{r}\sin\left(\phi \right)\cos\left(\theta \right) \end{array}$$

(4) $$\begin{array}{c} encoded\_v_{x}=\left\{\begin{array}{cc} 0, & \mathrm{if}\;v_{x}=0\\ \frac{0.5}{7}v_{x}, & \mathrm{if}\;0<v_{x}\leq 7\\ \frac{0.5}{18}v_{x}+0.3055, & \mathrm{if}\;7<v_{x}\leq 25\\ 1, & \mathrm{if}\;v_{x}>25\\ \frac{0.5}{7}v_{x}, & \mathrm{if}\;-7\leq v_{x}<0\\ \frac{0.5}{18}v_{x}-0.3055, & \mathrm{if}\;-25\leq v_{x}<-7\\ -1, & \mathrm{if}\;v_{x}<-25 \end{array}\right. \end{array}$$

This encoding is designed such that road users with slow speeds (like pedestrians, bicycles, etc.) up to 25 km/h (or approximately 7 m/s) are encoded in [−0.5, 0.5] and high-speed road users like cars, motorcycles, buses, etc., in the city area up to the speed of 90 km/h (or 25 m/s) are encoded in remaining half scale.

#### 4.2.3. Rendering of Projected Points to Solid Circles

The radar sensor used in this work provides around 500 points per frame within the overlapping field of view of the camera. However, the number of pixels in the black canvas used as the base for radar pseudo image generation is 2,334,720 pixels. Hence, projected and encoded radar points on the pseudo image cover only 0.0214% spatially, and the rest 99.978% is zero in all three channels. As a result, when such a pseudo image is fed into a deep learning network, it becomes difficult for the network to learn useful features from radar data.

(5) $$\begin{array}{c} radius=-\frac{x}{60}+4 \end{array}$$

To solve this, each projected and encoded radar point is rendered to a solid circle with the center point as the projected radar point pixel and radius calculated based on lateral distance *x* of the radar sensor using (5). In cases where two closely projected radar points overlap partially after rendering into a solid circle, then importance is given to the one that is close to the sensor, i.e., at a short distance from the sensor. Hence, the radar point close to the sensor is rendered complete, and another one is rendered partial (excluding the overlapping area).

It is worth noting that the function in Equation (5) is obtained after performing multiple experiments during the process of radar pseudo image generation. The main intention of selecting a proper rendering function is to logically reduce the black space of the radar pseudo image while avoiding unnecessary overlapping of points, especially in the far region. Hence, a visual approach is considered for finding an appropriate rendering function. For this purpose, different piece-wise linear as well as full-scale linear functions with different radius values are generated that are based on the distance of radar points from the sensor. Additionally, to preserve the projection effect in the 2D plane, nearby points are rendered with a large radius while far-away points are rendered with a relatively small radius. For each function, radar pseudo images for different frames (selected from the full dataset) are generated and visually analyzed so that points are well separated for most of the areas and also visually cover a sufficient amount of pseudo-image space.

**Figure 3:**
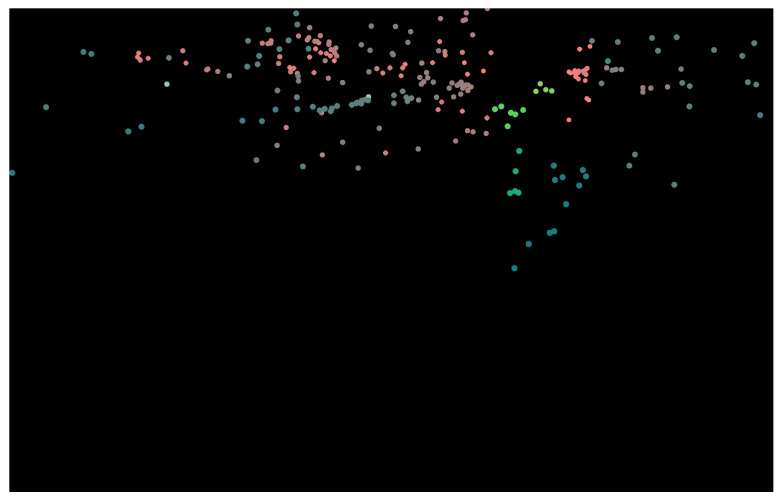
Three-channel radar pseudo image. For visualization purposes, encoded values in each channel from the range [−1, 1] are changed to [0, 255] linearly.

An example of a generated radar pseudo image using the stated process is shown in Figure 3. This image is represented in pixel coordinates. Further, as mentioned earlier, this pseudo image is formed by projecting the 3D radar points of one time frame to a 2D image-like pixel plane using the projection matrix. This projection matrix comprises the camera intrinsic matrix and the radar-to-camera extrinsic matrix that is obtained as per [38].

### 4.3. Camera Backbone

For the camera backbone, one of the most widely used deep learning architectures, known as ResNet (Residual Network) [39], is used. This architecture has 5 variants with 18, 34, 50, 101, and 152 layers, respectively. However, after experiments, ResNet with 18 layers is found sufficient and suitable for this work. The output from ResNet-18 is further combined with a feature pyramid network (FPN) [40] to generate multi-scale features as output from the camera backbone. The pre-trained model of ResNet-18 with FPN is readily available in the torch-vision library [42]; hence, it is used directly as a camera backbone for this work.

This backbone extracts features from the camera image at 4 different scales, named CP2, CP3, CP4, and CP5 in Figure 2. All these feature tensors comprises 256 channels each, but their spatial dimensions vary. Feature tensor CP2 is $\frac{1}{4}$ size, CP3 is $\frac{1}{8}$ size, CP4 is $\frac{1}{16}$ size, and CP5 is $\frac{1}{32}$ size of input image.

### 4.4. Radar Backbone

**Figure 4:**
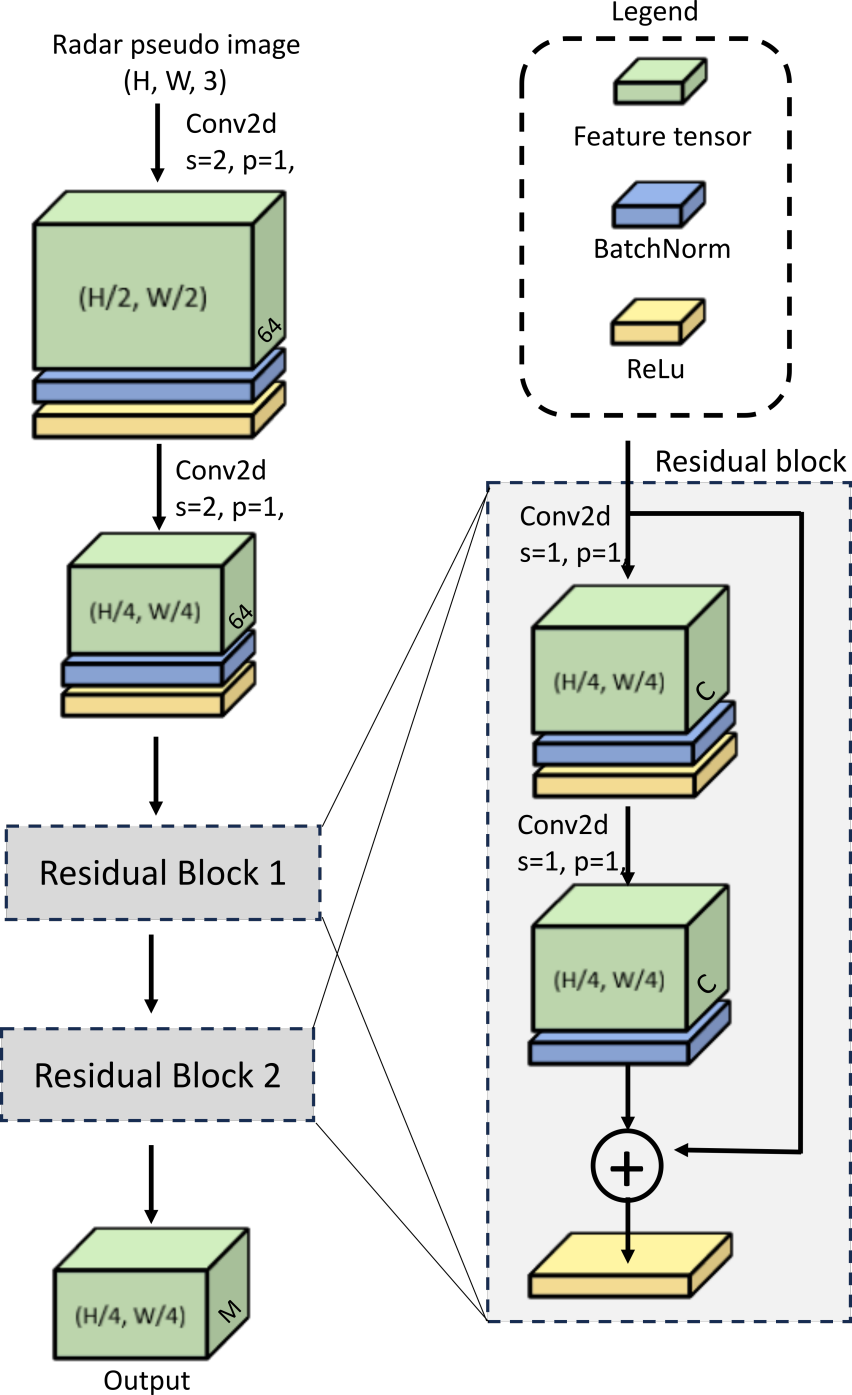
Radar backbone.

Compared to the camera image, the data in the radar pseudo image is much less; hence, for radar feature extraction, a custom small-size backbone architecture wwas designed for this work, as shown in Figure 4. In this backbone, at first, a 2D convolution layer with kernel size of 3×3, stride of 2, and padding of 1 is applied to the input radar pseudo image that results in feature tensor of size $\left(\frac{H}{2},\frac{W}{2},64\right)$ where *H* and *W* are the height and width of the radar pseudo image. Then, a batch-norm [43] and ReLu (rectified linear unit) activation function is applied on this feature tensor.

Then, again, the convolution layer with a kernel size of 3×3, stride of 2, and padding of 1 is applied resulting in further reduction in feature tensor to size of $\left(\frac{H}{4},\frac{W}{4},64\right)$ and then followed by batch-norm and relu. Thereafter, it is fed into residual block 1 [39] that generates the feature tensor of size $\left(\frac{H}{4},\frac{W}{4},128\right)$. For residual block 1, the value of parameter *C* is 128.

However, depending on the feature-merger strategy, the number of channels of the residual block 2 varies. If the radar and camera features are concatenated in the feature merger block then the output feature tensor from residual block 2 of the radar backbone is of size $\left(\frac{H}{4},\frac{W}{4},128\right)$ and value of C = 128 and M = 128 in Figure 4. If the radar and camera features are element-wise added in the feature merger block then the output feature tensor from residual block 2 of the radar backbone is of size $\left(\frac{H}{4},\frac{W}{4},256\right)$ and value of C = 256 and M = 256 in Figure 4.

### 4.5. Radar-Camera Feature Merger

**Figure 5:**
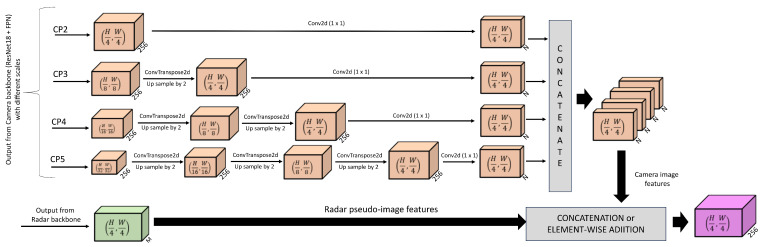
Radar–camera feature merger network.

The radar–camera feature merger network is shown in Figure 5, where the processing of multi-scale camera features is illustrated in a light red color and the radar features are illustrated in a green color for better visualization. In this work, the two most widely used techniques to merge features from different sensor modalities in deep learning models are used. These are element-wise addition and concatenation. Through these techniques, features from each sensor are mathematically integrated in a systematic and structured manner.

The camera backbone generates 4 feature tensors of different scales. These are named CP2, CP3, CP4, and CP5 as stated before. Among these, CP2 is the largest feature tensor with size $\left(\frac{H}{4},\frac{W}{4},256\right)$. Hence, apart from this feature tensor, all other feature tensors are up-sampled one or more times in multiples of 2 by using 2D transpose convolution layers to increase spatial dimensions to $\left(\frac{H}{4},\frac{W}{4},256\right)$. Then, all 4 feature tensors are fed into a 2D convolution layer with the kernel of size 1×1 to reduce each of their channels from 256 to *N*. This process of scaling different-sized features to one is partially inspired by the work of [30].

The value of *N* and *M* in Figure 5 depends on the feature merging strategy. If features from the camera and radar are concatenated, then the value of *N* is 32 and the value of *M* is 128. This results in individual feature tensors from the camera and radar with a size of $\left(\frac{H}{4},\frac{W}{4},128\right)$ and after feature concatenation, the merged feature tensor has a size of $\left(\frac{H}{4},\frac{W}{4},256\right)$. Similarly, if features from the camera and radar are added element-wise, then the value of *N* is 64 and the value of *M* is 256. This results in individual feature tensors from camera and radar of size $\left(\frac{H}{4},\frac{W}{4},256\right)$ and after element-wise addition of features, the merged feature tensor again has a size of $\left(\frac{H}{4},\frac{W}{4},256\right)$. Hence, irrespective of the feature merger method, the final size of the merged feature tensor remains the same.

### 4.6. Radar Point Cloud-Based Region Proposal Network

This part of the model comprises a region proposal head, a radar point cloud-based anchor generator, and post-processing of anchors to obtain proposals.

#### 4.6.1. Region Proposal Head

**Figure 6:**
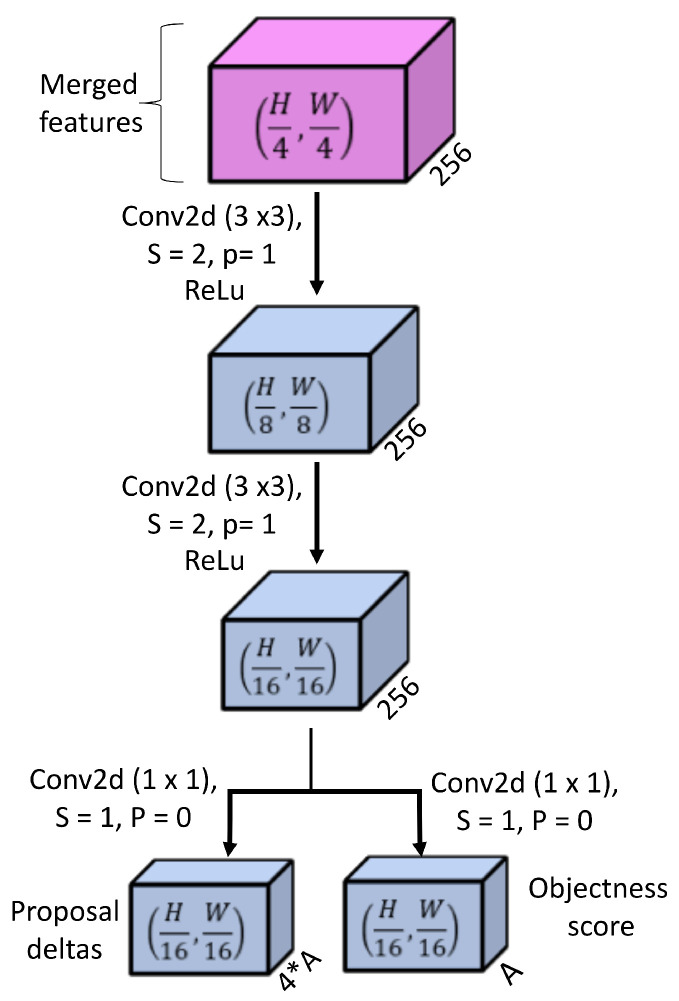
Region proposal head.

Radar camera merged feature tensor with a size of $\left(\frac{H}{4},\frac{W}{4},256\right)$ is given as input to the region proposal head. This head is a small neural network, as shown in Figure 6. It comprises two sequential operations of 2D convolution together with relu that results in an intermediate spatially reduced feature tensor with a size of $\left(\frac{H}{16},\frac{W}{16},256\right)$. Then, two separate 2D convolutions are applied to generate objectness score as tensor of logits of size $\left(\frac{H}{16},\frac{W}{16},A\right)$ and proposal deltas tensor of size $\left(\frac{H}{16},\frac{W}{16},A\times 4\right)$ where *A* is the total number of anchors generated per pixel at scale of $\frac{1}{16}$ of input size.

#### 4.6.2. Radar Point Cloud-Based Anchor Generator

In bad weather and light conditions, camera object detection degrades drastically, but radar generates one or more points from road users due to its robustness. This signifies the fact that if the radar gives valid points from some place within the field of view, then there are high chances of having a road user in that area, even if it is not sufficiently visible in the corresponding camera image. Hence, unlike state-of-the-art camera-based object detectors such as [44,45], where anchors are generated at every pixel, in this work, anchors are generated where the projected radar points are available in the radar pseudo image. The idea of generating anchors from radar points is inspired by the work of [16].

However, before generating anchors from each radar point, the noise points generated from the static environment in the smart infrastructure-based setup are filtered out to the maximum using the background subtraction technique as explained in Section 4 of [37]. Then, the leftover static foreground points and all the dynamic points are used to generate anchors. The information of dynamic and valid static points is preserved in memory during the generation of the radar pseudo-image. For each point, multiple anchors are generated with sizes [16, 32, 64,128, 256,512, 1024] pixels, aspect ratios [0.25, 0.5, 1, 2, 4] and different directions such as shifting in all four directions (top, left, right, bottom) by 25%, shifting in all four directions by 75%, shifting diagonally in all four directions (top left, bottom left, top right, bottom right) by 50%, and one kept in center. With all these combinations, 5×7×13=455 anchors are formed per radar point.

#### 4.6.3. Post-Processing of Anchors to Obtain the Proposals

The region proposal head generated proposal deltas and objectness scores as logits for every pixel of the feature tensor with a size of $\frac{1}{16}$. These logits are passed through the sigmoid activation function to convert them into objectness scores as probabilities. However, only values at pixels where the radar point is found are required. Hence, here indexing is performed where at first the (u,v) value of each radar point is scaled by $\frac{1}{16}$ and then its integer value is used to index the objectness score and deltas from the output of the region proposal head. The rest of the information about the head is discarded. The method of indexing is used because the number of radar points in every frame is not a fixed value, and hence, a fixed-size head is not possible to design in the region proposal directly.

After indexing, corresponding deltas are applied to each anchor to change their sizes, and then modified anchors are first clipped to the image size, and extremely small invalid anchors are removed. After this stage, the modified anchors are called proposals. Then, based on their corresponding objectness score obtained from the region proposal head, at first, very low score proposals are discarded, and then non-max suppression is applied to select the top *N* proposals. During the training phase, these proposals are further classified as foreground (fg) and background (bg) proposals with the help of ground truth data. During inference, there is no such classification that exists, and all the top *N* proposals are forwarded to the next stage of the model.

### 4.7. 2D Bounding Box and Class Head

The part of the model highlighted in yellow in Figure 2 is the bounding box and class head. The top *N* proposals generated from the radar point cloud-based region proposal network and radar–camera merged feature tensor are fed into this head. Generated proposals are in the scale of the input image size, while the merged feature tensor is in the scale of $\frac{1}{4}$ of the image size. The first stage in this head is a region of interest (ROI) align (refer to Section 3 of [46]) that generates a fixed-size tensor (10, 15, 256) for each proposal by applying the ROI alignment operation. This is then passed through 2D convolution, 2D maxpool, and ReLu to generate a feature tensor of size (5, 7, 256). This feature tensor is flattened and then passed through two linear neural networks with 1024 nodes each. The output of this is then passed through two independent linear layers to predict class logits of size (C,1) and 2D bounding box deltas with a size of (C,4) for each proposal. Here, *C* is the total number of object categories to predict. There are 5 object categories of road users in this work and one more category as background. So here, the value of *C* is 6.

In the post-processing part, the sigmoid function is applied to convert class logits into class probabilities, and values of deltas that correspond to the highest class probability are extracted and applied to the corresponding proposal to generate a 2D bounding box. This process is performed for all the top *N* proposals fed into this head. However, all these predicted bounding boxes are not valid for the final output. Hence, at first, all the bounding boxes with the class as background are removed, and then very low score (class probability) boxes are removed using the appropriate threshold value. Thereafter, the remaining bounding boxes are clipped to the input image size, and very small-sized bounding boxes are further discarded. At last, class-wise non-max suppression is applied where high overlapping bounding boxes with low class probability are removed, and then the maximum *K* bounding boxes with the highest score are given as output from this head. In this work, the value of *K* is selected as 100. This means the predicted output for one frame can provide a maximum of 100 detections (if available in the input).

### 4.8. Radar Segmentation Mask Head

Radar segmentation mask head separately predicts one or more radar points that belong to each road user. It is also referred to as instance segmentation of the radar point cloud in some of the literature. All the valid 2D bounding boxes along with their class that are obtained from the box and class head in the previous step are given as input to this head. The processing of this head is highlighted in blue in Figure 2.

Due to inherent errors during spatial calibration and time synchronization, all the radar points belonging to one object do not fall well within the 2D bounding box of the image plane after projection in all cases. Hence, to handle this, all the valid 2D bounding boxes are first expanded by a certain fraction and then clipped to the input image size. For each bounding box, the part of the radar-camera merged feature tensor is then cropped at $\frac{1}{4}$ scale. This results in a different feature tensor whose spatial dimensions are the same as the corresponding expanded bounding box. This is denoted as $\left(\frac{B_{h}}{4},\frac{B_{w}}{4},256\right)$ where $B_{h}$ and $B_{w}$ are height and width of the expanded bounding box, respectively.

This feature tensor of each proposal is then passed through a sequential 2D convolution with relu for 6 times using different numbers of kernels. This results in the number of output channels as [128, 64, 32, 16, 8, 2] in each subsequent operation and the final feature tensor of size $\left(\frac{B_{h}}{4},\frac{B_{w}}{4},2\right)$. The first channel of this output tensor is passed through the *sigmoid* function to obtain probabilities (in the range of [0, 1]) of the spatial position of projected radar points in the form of uv values. This is denoted as *uv mask* in Figure 2. The second channel of this feature tensor is passed through the *tanh* function to obtain corresponding radar point values of distance *x* encoded in range [−1, 1]. This is denoted as *r mask* in Figure 2.

These outputs are then fed into a post-processing block. Here, at first, a threshold is applied to the *uv mask* to extract only valid radar points for each bounding box. These valid radar points are then again checked with the encoded values of projected points from the input radar pseudo image to find which predicted points are available in the input data. The predicted points that pass the threshold of the *uv mask* but are still not found in the input data are also removed. Generally, when the 3D radar points are projected on the 2D image-like plane to generate the pseudo image, the depth information is lost. As a result, in some cases, when two radar points having different depths but very close spatial distances are mapped as one point on the projected plane. Hence, after finding the valid radar points, the corresponding values from predicted *r mask* are decoded to resolve such ambiguities. Kindly note that the encoded values of velocity are not predicted in the output because they are not required to segment the radar points. However, they play a crucial role in the process of feature generation and detection in the complete model.

At last, the list of all radar points predicted and processed for each bounding box is then given as output from this head.

### 4.9. Losses

Different loss functions are utilized for loss calculations. A total 6 individual losses and their weighted sum as total loss are calculated during the training. The first two losses are in the radar point cloud-based region proposal network that provides objectness scores and proposal delta values. For objectness score loss $L_{po}$, binary cross entropy with logits [47] is used, and for proposal delta loss $L_{pd}$, smooth L1 loss [48] is used. Similarly, two losses are calculated in the 2D bounding box and class head. For object classification loss $L_{cls}$, binary cross entropy [49] is used, and for 2D bounding box deltas $L_{bbox}$, again smooth L1 loss [48] is used.

In the radar segmentation mask head, *uv mask* $L_{uv}$ loss is calculated using binary cross entropy loss with logits [47]. Generally, the *uv mask* contains a lot of pixels where radar points do not exist in the ground truth. Hence, pixels where radar points exist are given more weight during ground truth mask generation. In this work, a value of 10 is assigned to such pixels, and other pixels are assigned a weight of 1. This helps to converge the loss function faster and also improves output accuracy. The loss for *r mask* $L_{r}$ loss is calculated via smooth L1 loss [48] for the pixels where radar points exist in the ground truth mask.

The total loss $L_{total}$ is a weighted sum of all the 6 losses as given in Equation (6). The values of weights in $L_{total}$ are based on experiments with the smart infrastructure-based sensor setup used in this work.

(6) $$\begin{array}{c} L_{total}=L_{po}+10L_{pd}+L_{cls}+50L_{bbox}+10L_{uv}+50L_{r} \end{array}$$

## 5. Experiments and Results

At first, different experiments are conducted by either changing certain hyperparameters of the proposed Infra-3DRC-FusionNet or by modifying certain parts to obtain optimum performance on the validation set. All these conducted experiments are listed in Table 3. For performance evaluation, at first, separate F1-scores are computed for IoU (intersection over union) thresholds of 0.5 and 0.75, and then the average F1-score is used to understand the performance of each variant.

During each experiment, the proposed model is trained for the same number of epochs with the same training and validation dataset to obtain a fair comparison of the performance. Table 4 lists the major hyperparameters used during the training of the model.

The best-performing model of Infra-3DRC-FusionNet (experiment 4 of Table 3) is then used for further evaluations using ground truth data (explained in Section 3.2). As stated before, the inference output of the Infra-3DRC-FusionNet generates a list of predictions where each prediction consists of four critical information about each detected road user. These include the location of the 2D bounding box in the image plane, one of the five object categories, the confidence score, and a list of all the associated radar points to the road user. This is highlighted as *output 1* in Figure 2. Associated radar points of each road user predicted by the model as *output 1* are then transformed to the ground plane using calibration, and then the center point is used to obtain the location of the road user in the 3D ground plane along with the associated object category and confidence score. In this paper, all the evaluations are performed on *output 1* and *output 2* of Figure 2. Details and evaluations of *output 3* of Figure 2 obtained from the 3D shape estimator will be presented and published separately.

For evaluation, at first, 3D-oriented bounding boxes of the lidar frame (ground truth data) are transformed to the ground plane of the smart infrastructure setup using extrinsic calibration. The corresponding predicted 3D point object from Infra-3DRC-FusionNet in the ground plane is associated using the inscribed (point-in-box) association metric. In this association, if the predicted 3D point object is inside the ground truth 3D bounding box, has the correct predicted object category, and has a confidence score higher than a given threshold, then it is considered a true positive. Otherwise false positive or a false negative is considered.

**Figure 7:**
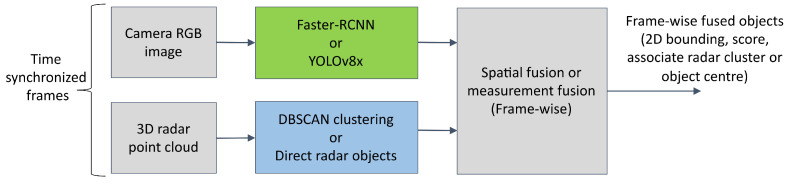
Object-level frame-wise spatial fusion or measurement fusion of camera-radar measurement (No tracking).

One of the main aims of the proposed deep fusion is to enhance the detection of road users (frame-wise object detection), especially in bad weather and light conditions where state-of-the-art decision-level fusion performance (frame-wise) often degrades. Hence, it is also important to evaluate and compare the output of decision-level fusion with the proposed deep fusion model using the same test data and ground truth labels. However, in general, the term object-level fusion refers to the fusion of data from two or more sensors using some form of filtering that generates track objects using the history of detection from previous frames (temporal fusion). But when two or more sensors can provide the data, almost at the same time (due to good time synchronization), it is possible to first generate a fused object (spatial fusion or measurement fusion) frame-wise and then this fused object can be fed into a tracking algorithm to estimate the states (temporal fusion). As the proposed deep fusion provides frame-wise output, to compare its performance with object-level fusion, only frame-wise spatial fusion is used [12]. The high-level pipeline of this process is illustrated in Figure 7. Furthermore, four different variants are used for object-level frame-wise spatial fusion by carefully selecting two state-of-the-art image-based object detectors and two radar-based object detectors. Faster-RCNN (faster region-convolutional neural network) and YOLOv8x (you only look once version eight extra large model) are used as image detectors. For radar-based object detection, the state-of-the-art, most widely used DBSCAN-based clustering and direct object list obtained from the sensor are used. It is worth noting that the object detection algorithm of the radar sensor from the manufacturer requires initialization by motion when deployed in vehicles. But in static infrastructure, this initialization is not possible, and results in some offsets in the object detection output. However, this offset is manually removed by examining the data and then used for comparison.

Unlike vehicle-based perception, infrastructure-based perception has a constant non-moving field of view (FoV) with a certain maximum distance. In this work, this maximum distance is considered as 80 m for cars and buses, and 50 m for VRUs (persons, bicycles, and motorcycles). It implies that within this field of view, a road user should be detected and recognized a maximum number of times correctly for corresponding ground truth for accurate and consistent perception.

Hence, the proposed deep fusion and four different combinations of object-level frame-wise spatial fusion are evaluated with the ground truth data from lidar using two groups of metrics. One group of metrics does scene-based evaluation, where for each type of road user, correct detection and recognition are checked with ground truth throughout its presence in the overlapping field of view of sensors, and then the average is calculated for all the instances of each road user for all the test scenes. This group of metrics helps to understand the performance of object detection algorithms in a temporal context. For example, let us consider that in a particular test scene (ground truth data), a road user has a lifespan of 100 frames in ground truth labels from its entry in the sensor field of view to its exit. The following metrics are defined as follows:

1. Mostly Detected (MD)—detected and classified correctly for 70% or more frames.
2. Partially Detected (PD)—detected and classified correctly for more than 50% but less than 70% of frames.
3. Partially Lost (PL)—detected and classified correctly for more than 20% but less than 50% of frames.
4. Mostly Lost (ML)—detected and classified correctly for less than 20% of frames.
5. $ED_{5}$—detected and classified correctly at least one time within first 5 consecutive frames of entry into sensor FoV.
6. $ED_{10}$—detected and classified correctly at least one time within first 10 consecutive frames of entry into sensor FoV.

Results of this evaluation are given in Table 5 and discussed later in this section. The second group of metrics evaluates the performance of deep fusion and object-level frame-wise spatial fusion for all the scenes by calculating precision, recall, and F1-score values. These are documented in Table 6.

### Discussions

Before discussing the results of experiments, it is important to note that the proposed Infra-3DRC-FusionNet is an object detection algorithm and generates only frame-wise output. It should not be misunderstood with track objects, where output is determined by using the history of objects from previous frames. In the general perception pipeline, the objects generated by Infra-3DRC-FusionNet can be fed into a tracking algorithm, if required, but in this paper, the tracking algorithm is not the main focus and is not part of this work.

Now, as highlighted in Table 3, Experiments 1, 2, and 6 have been conducted using the radar–camera feature concatenation approach, while Experiments 3, 4, and 5 have used the radar–camera feature element-wise addition approach. Moreover, Experiments 1 to 6 have been conducted using separate backbones for camera and radar feature extraction (commonly referred to as feature-level fusion) as highlighted in Figure 2, but in Experiments 7 and 8 all the six channels of camera and radar data are fed together into one ResNet18 backbone via one residual block. This is commonly referred to as data-level fusion. The last two experiments aimed to understand whether a single backbone approach is better than a separate backbone approach for deep fusion. It is evident from the average F1-score that a separate backbone approach has almost 10% better results compared to a common backbone approach.

From Experiments 1 and 2, one can conclude that re-training all 5 layers of ResNet18 of the camera backbone yields higher performance than re-training only the last three layers. Experiments 3 and 4 signify that using weights to balance the object class distribution helps to improve the performance by 0.4% when element-wise addition is used to merge camera and radar features. However, adding weights to balance the class for the feature concatenation approach in Experiment 6 has a bit lower performance than Experiment 3. Further, in Experiment 5, an Adam (adaptive moment estimation) optimizer [50] with a learning rate of 0.001 has lower performance compared to SGD (stochastic gradient descent) with a learning rate of 0.01 in other experiments. As a conclusion, Experiment 4 provides the best performance on the validation set.

Table 5 provides road user category-wise performance in a temporal context. The value of MD (mostly detected) for all five road users is high for the proposed deep fusion methodology as compared to all the four variants of the object-level frame-wise spatial fusion. It implies that the output of deep fusion generates more consistent frame-wise object detection, and can help to track road users (if a tracking algorithm is implemented) in bad light and weather conditions using camera and radar sensors more effectively. Additionally, values of $ED_{5}$ and $ED_{10}$ are also high in deep fusion for all road user categories. This implies that deep fusion can also help to initialize the track faster (if a tracking algorithm is implemented) by conducting early and correct object detection when a road user enters the field of view of the smart infrastructure sensor setup.

Similarly, results given in Table 6 signify that the proposed deep fusion approach has very high performance as compared to all the four variants of the object-level frame-wise spatial fusion (commonly referred to as late fusion) on the complete test set. Deep fusion has a precision of 0.92 and a recall of 0.78. This results in a total F1-score of 0.85 in the 3D ground plane on the complete test set. It has 33%, 6%, and 21% improvement in precision, recall, and F1-score, respectively, as compared to top-performing object-level frame-wise spatial fusion output (with Faster-RCNN and DBSCAN variant).

Hence, fusing radar and camera data at an early-stage, especially after extracting some intermediate features from each sensor and fusing them using deep neural networks enhances the overall object detection performance in different light and weather conditions for smart infrastructure-based setup.

**Figure 8:**
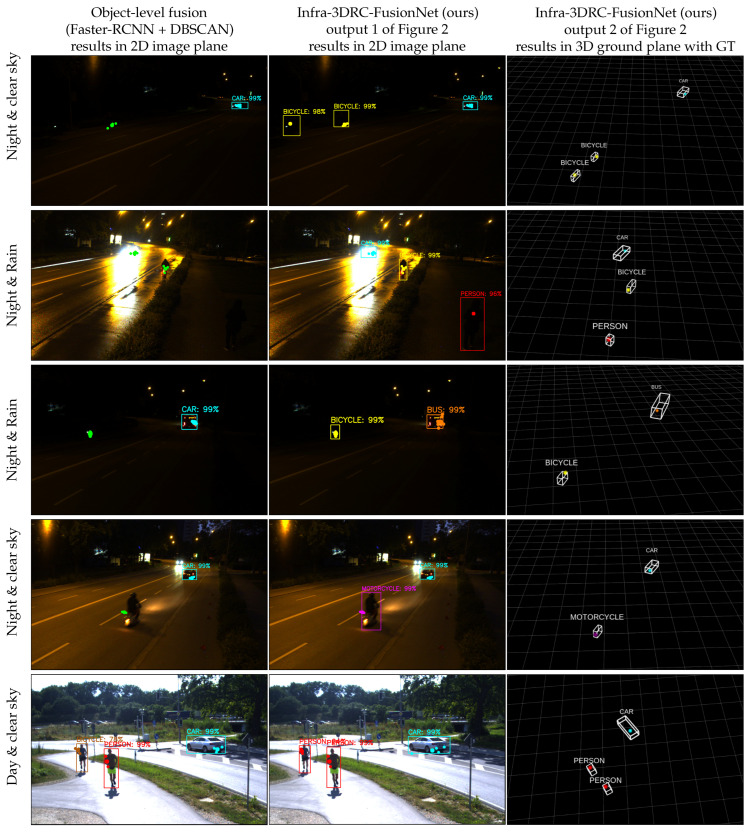
Each row corresponds to one sample output with different weather and/or light conditions and different types of road users. Column-wise, the first column shows the output from object-level frame-wise spatial fusion in the image plane for the best-performing variant (Faster-RCNN and DBSCAN). The second column shows output using Infra-3DRC-FusionNet in the image plane, and the third column shows 3D point object output from Infra-3DRC-FusionNet in the ground plane along with corresponding 3D ground truth bounding boxes.

Figure 8 shows some example results where the first column shows the output from object-level frame-wise spatial fusion (Faster-RCNN and DBSCAN variant) in an image plan with 2D bounding boxes, object category, confidence score, and associated radar cluster points. The green points without a bounding box and class are those radar cluster points that are not detected by the camera due to either low light or bad weather. The second column shows the results of Infra-3DRC-FusionNet in the image plane, where each detected object is highlighted with a 2D bounding box, object category name, confidence score (in %), and all the associated radar points per object predicted by the network. This is output 1 of the Infra-3DRC-FusionNet as shown in Figure 2. The third column shows output 2 of the Infra-3DRC-FusionNet where each detected road user is transformed as a 3D point object in the ground plane and then the corresponding ground truth 3D bounding box (from the lidar sensor) is also shown to validate that the predicted 3D point is well within each ground truth box. This output (in column 3) is generated using a black background in Rviz (ROS visualization tool), and it does not show the actual daylight situation of the collected data.

In the first row, two bicycles are correctly detected and classified by deep fusion during the night, while in object-level spatial fusion, one bicycle is not detected by both sensors, and another is not detected by the camera. In the second row, the car is not detected in the object-level spatial fusion by the camera due to a high reflection of the headlight from a wet road surface. Also, the VRUs (both bicycle and person) are not detected by the camera due to rain and dark environments. But by deep fusion, all the road users are well detected and classified correctly. Similarly, in the third row, in object-level spatial fusion, the bicycle is not detected by the camera, and a bus is misclassified as a car. However, deep fusion detects and correctly classifies both the traffic users, even in a very dark environment. Further, in the fourth row, the detection of the motorcycle is correct in deep fusion. In the Fifth row, one person is misclassified as a bicycle in the object-level spatial fusion, but it is correctly detected and classified by deep fusion. With these sample outputs, it is evident that deep fusion provides enhanced object detection and classification even in adverse environmental conditions.

**Figure 9:**
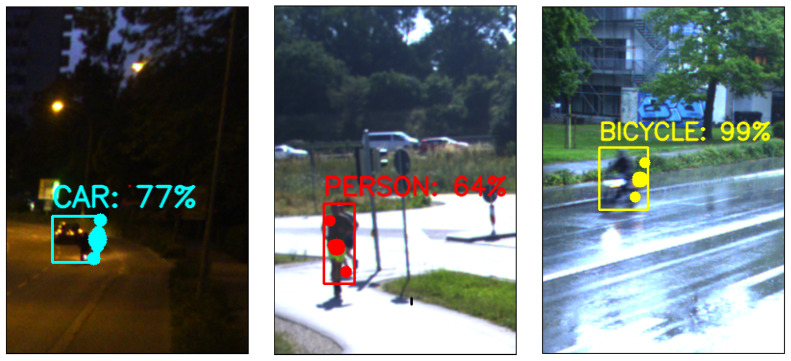
Sample cases with incorrect detections by proposed methodology.

In Table 5, the value of mostly detected bicycles is 0.67 means 67%, and partly detected bicycles is 0.22, i.e., 22%. It implies that some more improvements in bicycle detection are still necessary so that cases of partially detected bicycles become shifted to mostly detected. Similarly, motorcycle detection also needs improvement as currently only 25% of them are mostly detected. Figure 9 depicts some cases of incorrect detections by the proposed deep fusion. In the first two cases, it happens due to occlusion, and in the last case, the motorcycle is incorrectly classified as a bicycle. Future work will focus on improving the detection quality of bicycles and motorcycles. Finally, with all the conducted experiments and evaluations on output 1 and output 2 of the proposed Infra-3DRC-FusionNet, it is successfully proven that in different light and weather conditions, fusing mono camera and 3D automotive radar data at an early-stage results in very high frame-wise object detection performance.

## 6. Conclusions

In this work, a novel deep fusion methodology along with the model architecture (named Infra-3DRC-FusionNet) to fuse the RGB image data of a mono-camera and 3D automotive radar point cloud data for a roadside-mounted smart infrastructure-based sensor setup is described. Details of the measurement setup, data collection, annotation, and description of each part of the model are provided. The proposed methodology generates enhanced object detection in different light and weather conditions at three levels. These are in the 2D image plane, 3D point objects in the ground plane, and 3D extended objects in the ground plane. In this paper, evaluations were performed on the first two levels, and the third level will be presented and published later. Various experiments are conducted on the developed deep fusion model with different settings, and the best-performing model is then evaluated using ground truth data of the lidar sensor (also referred to as the test set). The test data consists of various scenes recorded in different light and weather conditions. It results in a precision of 92%, recall of 78%, and F1-score of 85% for all the five road user categories on the complete test set for predicted 3D point objects (along with object category and confidence score) in the ground plane of the smart infrastructure setup. Additionally, the described deep fusion methodology is benchmarked with four different variants of decision-level fusion on the complete test set. It provides an improvement in precision, recall, and F1-score of 33%, 6%, and 21%, respectively, when compared with the best performing object-level spatial fusion variant (Faster-RCNN + DBSCAN). Further improvement of bicycle and motorcycle detection is part of the future work.

## Figures and Tables

**Table 1:** Statistics of training and validation set.

Set	Person	Bicycle	Motorcycle	Car	Bus	Frames
train: easy	670	1772	1151	3634	539	3232
train: difficult	382	1259	277	1429	418	2137
train: ∑	1052	3031	1428	5063	957	5369
val: easy	305	694	406	1591	226	1310
val: difficult	143	548	104	588	197	896
val: ∑	448	1242	510	2179	423	2206
train + val ∑	1500	4273	1938	7242	1380	7575

**Table 2:** Statistics of the ground truth data (also referred to as test data).

Environment	Scenes	Person	Bicycle	Motorcycle	Car	Bus	Frames
Night + clear sky	7	0	323	51	566	174	651
Night + Rain	4	285	329	0	196	0	360
Day + Clear sky	7	579	577	0	547	0	719
Day + Rain	10	959	504	53	627	82	921
∑	28	1823	1733	104	1936	256	2651

**Table 3:** Performance of various experiments conducted on the proposed Infra-3DRC-FusionNet on the validation set. F1-score is an average of IoU of 0.5 and 0.75.

Exp No.	Description	Avg F1-Score
1	separate backbones for the camera and radar	0.8777
	SGD (0.01), feature concatenation	
	ResNet18 only last three layers re-trained	
2	separate backbones for the camera and radar	0.8889
	SGD (0.01), feature concatenation	
	ResNet18 all five layers re-trained	
3	separate backbones for the camera and radar	0.8896
	SGD (0.01), feature element-wise addition	
	ResNet18 all five layers re-trained	
4	separate backbones for the camera and radar	0.8934
	SGD (0.01), feature element-wise addition	
	ResNet18 all five layers re-trained	
	class balanced by adding weights	
5	separate backbones for camera and radar	0.6498
	Adam (0.001), feature element-wise addition	
	ResNet18 all five layers re-trained	
	class balanced by adding weights	
6	separate backbones for camera and radar	0.8833
	SGD (0.01), feature concatenation	
	ResNet18 all five layers re-trained	
	class balanced by adding weights	
7	One backbone Residual block with resNet18	0.7906
	All six input channels stacked together	
	SGD(0.01)	
	ResNet18 only last three layers re-trained	
8	One backbone Residual block with resNet18	0.7999
	All 6 input channels stacked together	
	SGD (0.01), ResNet18 all five layers re-trained	
	class balanced by adding weights	

**Table 4:** Major hyper-parameters used for the training.

Hyper-Parameter	Value
Batch size	4
Optimizer	SGD
Learning rate	0.01
Gradient clipping	10
LR Schedular	CosineAnnealingLR
Epochs	100
classes	5
camera image size (h, w, c)	(1216, 1920, 3)
radar pseudo image size (h, w, c)	(1216, 1920, 3)
Proposal Expansion fraction	0.15
uv binarization threshold	0.7
feature merging	Concatenation/element-wise addition
camera feature upsampling	TransposeConv

**Table 5:** Comparison of performance of proposed deep fusion with four variants of the object-level frame-wise spatial fusion on test data.

Object Type	MD(↑)	PD	PL	ML(↓)	${\mathrm{ED}}_{5}\left(↑\right)$	${\mathrm{ED}}_{10}\left(↑\right)$
**Object-level fusion—Faster-RCNN and DBSCAN**						
person	0.84	0.10	0.05	0.0	0.89	1.0
bicycle	0.30	0.11	0.3	0.30	0.63	0.70
motorcycle	0.0	0.0	0.0	1.0	0.0	0.25
car	0.91	0.05	0.03	0.0	0.92	0.95
bus	0.83	0.0	0.17	0.0	1.0	1.0
**Object-level fusion—YOLOv8x and DBSCAN**						
person	0.47	0.21	0.32	0.0	0.58	0.68
bicycle	0.19	0.04	0.15	0.63	0.48	0.55
motorcycle	0.0	0.0	0.0	1.0	0.0	0.0
car	0.81	0.03	0.14	0.03	0.86	0.89
bus	0.67	0.0	0.17	0.17	0.83	0.83
**Object-level fusion—Faster-RCNN and Radar objects**						
person	0.05	0.05	0.05	0.84	0.16	0.16
bicycle	0.51	0.18	0.22	0.07	0.44	0.48
motorcycle	0.0	0.75	0.25	0.0	0.25	0.75
car	0.89	0.10	0.11	0.0	0.51	0.78
bus	0.67	0.17	0.17	0.0	0.83	0.83
**Object-level fusion—YOLOv8x and Radar objects**						
person	0.05	0.0	0.11	0.84	0.05	0.05
bicycle	0.48	0.11	0.3	0.11	0.33	0.41
motorcycle	0.0	0.50	0.5	0.0	0.50	0.75
car	0.76	0.16	0.08	0.0	0.51	0.75
bus	0.83	0.0	0.17	0.0	0.83	0.83
**Deep fusion (ours)**						
person	0.90	0.05	0.05	0.0	0.95	0.95
bicycle	0.67	0.22	0.11	0.0	0.96	1.0
motorcycle	0.25	0.25	0.0	0.5	0.75	0.75
car	0.92	0.0	0.08	0.0	0.97	1.0
bus	1.0	0.0	0.0	0.0	1.0	1.0

**Table 6:** Benchmarking results with high-level object-level fusion.

Fusion Type	Precision	Recall	F1-Score
FasterRCNN and DBSCAN	0.59	0.72	0.64
YOLOv8x and DBSCAN	0.47	0.58	0.52
FasterRCNN and radar objects	0.29	0.53	0.37
YOLOv8x and radar objects	0.27	0.50	0.35
**deep fusion (ours)**	**0.92**	**0.78**	**0.85**

## Data Availability

A subset of the dataset used to train the model in this work is available at https://github.com/FraunhoferIVI/INFRA-3DRC-Dataset, accessed on 1 April 2025 [51]. The full dataset used in this article is not readily available because of the ongoing research.
